# Effects of Spice Mixtures and Konjac Glucomannan–Whey Protein Isolate Based Edible Films on the Microbiological Stability and Textural Properties of Cheese

**DOI:** 10.3390/foods14223819

**Published:** 2025-11-07

**Authors:** Aslı Çelikel Güngör, Mutlu Buket Akın, Emir Ayşe Özer

**Affiliations:** 1 Department of Gastronomy and Culinary Arts, Faculty of Tourism, Mardin Artuklu University, Mardin 47200, Turkey; 2 Department of Food Engineering, Faculty of Engineering, Harran University, Şanlıurfa 63300, Turkey; 3 Department of Food Engineering, Faculty of Agriculture, Hatay Mustafa Kemal University, Hatay 31060, Turkey

**Keywords:** cheese, edible film, konjac glucomannan, optimize, spices, whey protein isolate

## Abstract

Some of the main factors affecting the product quality and shelf life of cheese include weight loss during storage, microbial spoilage, and structural changes in processing technology. Edible films are coating materials produced with the aim of improving quality properties and extending shelf life, and various studies have been conducted on their properties. However, research examining the effects of coatings enriched with spice-derived natural antimicrobial compounds is scarce in relation to cheese quality. In this study, edible films and spice mixtures were applied to cheese during storage, and their effects on weight loss, textural properties, and microbiological stability were investigated. Response surface methodology was used to determine the edible films and spice mixtures used in the coatings. The amounts of whey protein isolate and konjac glucomannan in the composition of edible films were found to affect water vapor permeability, elongation coefficient, and tensile strength properties; in the spice mixtures, thyme, rosemary, and red pepper were found to have antifungal effects. Based on the data obtained, it was determined that applying an edible film coating to the cheese surface reduced weight loss and improved textural properties, while applying a spice mixture coating increased microbial stability. This study demonstrates that the use of edible films supported by natural protective components could be a practically applicable, innovative, and sustainable approach to improving the properties of cheese and extending its shelf life.

## 1. Introduction

Food packaging provides a physical barrier against environmental factors such as moisture, oxygen, and microbial contamination, improving product safety, quality, and shelf life. Cheese storage processes are associated with significant quality losses, including surface mold growth and textural deterioration [1,2,3,4,5,6]; the microbiological spoilage caused by mold can prevent product consumption, leading to economic losses, and can also pose a risk to human health due to the toxic compounds produced by molds [7]. Additionally, moisture loss during storage occurs as water evaporates from the surface of the cheese and ambient humidity decreases, resulting in diminished product weight and textural properties [8,9]. In turn, this negatively affects the physical appearance, taste, and aroma of the cheese [10] and leads to textural hardening, loss of elasticity, and reduced cuttability [11]. The combination of these factors creates critical challenges that need to be managed during the storage process, significantly impacting both the sensory and economic value of the cheese [12]. Studies have shown that edible film coatings can limit moisture loss by forming a semi-permeable barrier on the product surface, slowing down microbial growth and reducing textural changes. These biodegradable films also stand out as an environmentally friendly alternative to traditional plastic packaging [13,14,15].

Edible films are active packaging systems that reduce the permeability of water vapor and gas by coating the food surface and can also feature antimicrobial and antioxidant components [16,17,18]. These films can extend shelf life by limiting water loss from food, suppressing microbial growth, and preserving nutritional value [19,20]. They are often composed of natural biopolymers such as carbohydrates, lipids, and proteins [21]. Among these components, polysaccharides are widely preferred due to their low cost, biocompatibility, and low gas permeability [22,23,24].

Konjac glucomannan (KG), obtained from the tuber of *Amorphophallus konjac*, is a polysaccharide with good film-forming capacity due to its β-1,4 and β-1,6-glycosyl bonds formed by D-mannose and D-glucose units [25]. However, due to their lack of hydrophilic structure, KG films alone demonstrate disadvantages such as low mechanical strength, poor water resistance, and limited antibacterial effects [26,27] and should be supported by other biopolymers to develop functional and durable film systems. Whey protein isolate (WPI) has shown significant contributions in this regard by improving both mechanical strength and gas barrier properties [28,29,30,31,32,33,34]; the hydrogen bonds formed between the carbonyl–amine groups in protein lysine–serine and hydroxyl groups in KG allow the film matrix to become more compact and increase its tensile strength [35]. Additionally, the interaction of KG and WPI molecules creates a filler-and-binder structure and decreases water vapor permeability. However, the disulfide, hydrogen, and hydrophobic bonds formed during WPI-based film production, along with denaturation, increase the tendency toward brittleness [36]. The addition of plasticizers such as glycerol in the production of these films both reduces brittleness and improves the elongation coefficient [34]. In addition, incorporating natural antimicrobial components in KG- and WPI-based edible films can strengthen their physical protection. In this context, spices stand out as natural preservatives that inhibit bacteria and molds due to the phenolic compounds they contain [30,37,38,39,40,41,42].

Spices are widely used in the food industry; some of their components have antibacterial and antifungal effects on various microorganisms. In vitro studies have shown that p-cymene, thymol, and 1,8-cineole, which are components of thyme, have an antifungal effect against *Aspergillus flavus* PTCC 5006, *Aspergillus niger* PTCC 5010, *Mucor himalis* PTCC 5292, and *Penicillium* spp. [41,42]. Ferdes et al. [39] determined that rosemary has an antifungal effect against *Mucor pusillus* ATCC 16458. Additionally, in vitro studies on rosemary and red pepper have shown that these spices also have inhibitory effects against various bacteria and fungi [37,38,40]. It is believed that these three spices can be used as functional ingredients in edible film formulations due to their broad-spectrum antimicrobial effects.

In this study, we aimed to develop a multifunctional edible film system that combines the natural antimicrobial components found in spices and the film-forming properties of KG and WPI and to determine its effect on cheese. There is no comprehensive study in the existing literature that improves on the low mechanical strength of KG, optimizes the barrier properties of WPI, or explores the microbial control potential of spices. In this respect, this research is expected to fill a significant gap in the field by providing a sustainable solution to fundamental problems such as moisture loss, mold growth, and texture deterioration during cheese storage.

## 2. Materials and Methods

### 2.1. Materials

We used fresh cow’s milk, food-grade melting salt, and CaCl_2_ (98–100% analytical purity, Merck KGaA, Darmstadt, Germany) for cheese production. Edible films were prepared using WPI (90% protein, Davisco Foods International, Inc., Le Sueur, MN, USA), KG (Mw ~200,000–2,000,000, Yunnan Gengyun Konjac Resources Developing Co., Kunming, China), glycerol (purity ≥ 99.5%), and antifoam A (A6582, 100% active silicone polymer, Sigma-Aldrich Co., St. Louis, MO, USA). Thyme (*Thymus vulgaris* L.), rosemary (*Rosmarinus officinalis* L.), and red pepper (*Capsicum tetragonum M*.) used in spice optimization were sterilized with gamma rays. The antifungal properties of the spices were determined using *Aspergillus niger* obtained from the Yıldız Technical University Microbiological Culture Collection, *Mucor* spp. from the Çukurova University Department of Food Engineering Culture Collection, and *Penicilium* spp. from the Harran University Department of Food Engineering Culture Collection. Potato dextrose agar (PDA—microbiological grade, Merck KGaA, Darmstadt, Germany), plate count agar (PCA—microbiological grade, Merck KGaA, Darmstadt, Germany), Man–Rogosa–Sharpe agar (MRS—microbiological grade, Merck KGaA, Darmstadt, Germany), and Elliker agar (Merck KGaA, Darmstadt, Germany) were used in the microbiological analyses. All other chemicals used in the study were of analytical purity.

### 2.2. Methods

**Figure 1:**
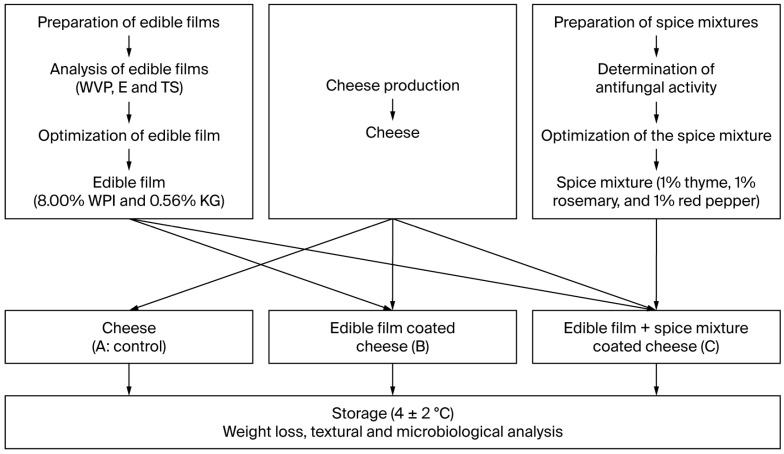
Edible films production flow diagram.

In the first phase of this study, edible films and spice mixtures containing WPI-KG were produced using the response surface method. Then, the edible film coatings and spice mixtures were applied to the surfaces of cheeses and analyzed during storage (Figure 1).

#### 2.2.1. Edible Film Production and Analysis

This section covers the production of the edible films, the optimization processes applied, and the chemical analyses performed within the scope of optimization.

##### Experimental Design and Optimization of Edible Film Production

We adhered to Central Composite Experimental Design and the response surface method to prepare films using Design Expert 6.02 software. Two independent variables were selected, 6–8% (*w*/*v*) for WPI and 0.4–0.7% (*w*/*v*) for KG, and pretrials were conducted to determine their ranges. The optimum mixture model, as described by Awolu et al. [43], was employed, and a regression equation was used to demonstrate the relationship between variables [44]. During the optimization, the effect of independent variables on the responses was analyzed using desirability functions. Individual desirability values ranging from 0 to 1 were defined for each response, and the optimal film formulation was determined by maximizing the overall desirability value. The coded levels and experimental points, along with the measured responses, are summarized in Table 1.

##### Edible Film Production

WPI and KG in the quantities specified in Table 1 were added to pure water (25 °C) to create total solutions of 100 mL each and then mixed (IKA, C Mag HS 7, Staufen im Breisgau, Germany) for 75 min. The mixtures were then heated at 90 °C in a water bath (NSB, Ankara, Turkey) for 30 min [26], then cooled down (22–25 °C) and mixed (1000 rpm, 15 min.) with 16% glycerin and 1–2 drops of antifoam. Next, 20 mL of edible film solution was placed in a 12 cm diameter glass Petri dish to dry at 50 °C for 48 h (moisture content: 16 ± 1%). The resulting films were subsequently analyzed for water vapor permeability (WVP), tensile strength (TS), and elongation (E) [45,46]. The analysis results applied to edible films are given in Table 2.

##### Water Vapor Permeability

Delrin cups were prepared according to the ASTM E96-92 standard. The WVP values of the films were determined gravimetrically [47]. Edible films were sealed over Delrin cups filled with anhydrous calcium chloride and placed in a desiccator containing saturated magnesium nitrate solution (53 ± 2% relative humidity, 25 °C). The pressure difference caused by the difference in internal and external relative humidity was 1,750 kPa. The desiccator was kept at 25 °C, and the weight loss of the containers was calculated by weighing them at 4-h intervals for 72 h using a scale with a sensitivity of ±0.0001 g. The water vapor permeability of the films/coatings was calculated with the following equation (Equation (1)) [48,49,50]. The measurements were carried out in triplicate.

P = (slope × x)/(A × Δp)(1)

P: Permeability (G mm/m^2^·d·kPa)
Δp: Partial pressure difference in gases
x: Film thickness (mm)
A: Surface area (m^2^)

##### Tensile Strength and Elongation Coefficient

The ASTM D638 standard was taken as the basis for determining the mechanical properties of the films [51]. The films were cut into 80 × 25 mm samples with the help of a mold prepared according to the standard and kept at 25 °C and 53 ± 2% RH for 48 h. The tensile strength and percentage elongation of the samples were determined using a TA-XT2 model mechanical testing machine (Stable Micro Systems, Surrey, UK). The tensile speed between the two jaws of the device was set at 1 mm/s. The maximum force and elongation applied to the sample at the breaking point were calculated using a computer program (Texture Expert Exceed 2.3, Stable Micro System, Survey, UK) connected to the mechanical testing device.

Stress was calculated by dividing the force applied to the sample at the fracture point by the initial cross-sectional area of the sample (N/mm^2^), and the percentage of elongation was calculated by dividing the change in sample length by the initial length (Equation (2)) [50]. The analysis was performed in triplicate.

% Elongation = ((L − L_0_)/L_0_) × 100 (2)

L_0_: Initial length of films
L: Final length of films

##### Microstructure of Edible Films

The microstructures of the films were determined by using scanning electron microscopy (SEM). To examine the optimized film structure, edible films were produced containing KG (0.70% *w*/*v*) and WPI (8.00% *w*/*v*), representing the upper limit for optimization. SEM analyses were performed with a Zeiss Evo-brand scanning electron microscope (EVO 10, Oberkochen, Germany) at voltages ranging from 5 to 15 kV [52].

#### 2.2.2. Spice Mixture Production and Analysis

This section presents our analysis of the optimization and antifungal effects of spice mixtures.

##### Experimental Design and Optimization of the Spice Mixture

A second mixture design was performed to optimize the ratios of thyme, rosemary, and red pepper extracts for antifungal activity (Table 3). Firstly, the lower and upper limits of the spices used for optimization were determined. In order to determine these limits, a preliminary study was conducted, and sensorially acceptable amounts of spice were determined. The proportions of spices (independent variables) to be used in edible film production were determined using Design-Expert 6.0 software (Stat-Ease Inc., Minneapolis, MN, USA). The spice blend with the highest antifungal effect was optimized based on desirability functions using the response surface technique. For each response, individual desirability functions were defined within the range of 0–1, and the overall desirability was calculated from these individual values using a weighted geometric mean [44]. The spice blend obtained from the optimization was selected based on the highest overall desirability (D) value, and this blend was used to coat the cheeses.

##### Preparation of Spice Mixtures

The spices were supplied ground and irradiated. The spice mixtures to be used in distillation experiments were weighed according to the values given in Table 3. In order to optimize the spice mixture, two different distillation methods were used: Soxhlet and hydrodistillation. In Soxhlet extraction (SER148/6, VELP Scientific, Usmate, Italy), the spice mixture was extracted with methanol in the Soxhlet apparatus. The spice and methanol were mixed in a 1:10 ratio and extracted at 55–60 °C for 6 h. Subsequently, the methanol in the mixture was removed under vacuum at 40–45 °C using a rotary evaporator (EV-3000 Series, Torontech Inc. Toronto, Canada). The obtained extract was stored at +4 °C for use in analysis [53]. For the hydrodistillation method, 100 g of the spice mixture was subjected to water distillation with a Clevenger apparatus for 3 h. The water of the essential oils was removed with anhydrous sodium sulfate and stored in a refrigerator (+4 °C) in dark, tightly closed bottles until use [54]. The obtained extracts and essential oils were mixed in a 1:1 ratio. Then, the inhibition zones generated by these mixtures against *Aspergillus niger*, *Penicillium* spp., and *Mucor* spp. were determined. The specified zoom diameters for the spice mixture are given in Table 4.

##### Preparation of Mold Spores and Determination of Antifungal Effect

In order to prepare the mold spores for study, *Aspergillus niger*, *Penicillium* spp., and *Mucor* spp. stock cultures were inoculated onto potato dextrose agar (PDA) using the slant agar method, and the molds were incubated at 25 °C for 3–5 days. Phosphate-buffered solution containing 5 mL sterile 0.05% Tween 80 was added to the slant agar under aseptic conditions in order to separate the spores of each mold type, and micelles were loosened slowly using sterile extract. The solution containing mold micelles and spores was then filtered through sterilized gauze and a glass funnel. Dilutions were prepared from the mold spores remaining at the bottom, and a dilution of 10^2^ CFU/mL was determined using the spread plate method on PDA [55].

The antifungal effect of plant extracts was determined using the “Agar Well Diffusion” method. Elliker agar was used to observe the growth of *Aspergillus niger* and *Penicillium* spp. First, 10 mL of Elliker agar containing 9 g/L agar was poured into Petri dishes, and the medium was allowed to solidify. Then, 10 mL of Elliker Agar containing 6 g/L agar was added to the solidified culture medium, and it was allowed to solidify again. The culture medium was inoculated with 0.1 mL of mold dilution (10^2^ CFU/mL) using the spread plate method. Later, a well with a diameter of 10 mm was drilled in the culture medium, and spice extract (0.2 mL) was added to the wells. The same procedure was performed for *Mucor* spp. using PDA instead of Elliker Agar. All prepared culture media were left for incubation (3–5 days at 25 ± 2 °C). Zone and mycelial formation in Petri dishes were observed daily, and the resulting inhibition zones were measured using a caliper [55].

#### 2.2.3. Cheese Production and Analysis

This section covers cheese production and the applied analyses.

##### Production and Coating of Sliceable Processed Cheese

Cheese production was carried out in accordance with the Turkish Food Codex Cheese Regulation 2015/6 [56]. Raw milk was heat-treated at 60 °C for 1 min, then rennet was added and left to coagulate at 32 °C for 40 min. The resulting curd was cut and left for 15 min. Then, 1/3 of the whey was removed, and the curd was heated to 40 °C while stirring gently. The curd was divided into blocks and left for fermentation at room temperature until its pH value reached 5.0. Once fermentation was complete, it was cut into pieces and dry-boiled (5 min at 70 °C). The curd was placed into molds after a smooth structure was observed and kept there for 2 days at 15 ± 2 °C. The cheeses were taken out of the molds and subjected to the coating process. It was determined that the sliceable processed cheese produced within the scope of this study had a dry matter content of 55.15%, a fat content of 23.83%, a protein content of 25.54%, and an ash content of 5.22%.

**Figure 2:**
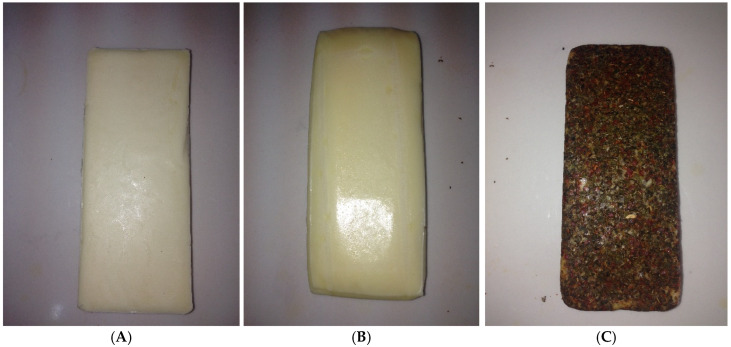
Images of the cheeses produced within the scope of the study. (**A**) control, (**B**) edible-film-coated cheese, and (**C**) edible film + spice mixture-coated cheese.

The produced cheeses were divided into three groups for coating. No coating was applied to the first group (A: control), only edible film was applied to the second group (B), and both edible film and the spice mixture were applied to the third group (C). The dipping method was applied for coating the edible film on the cheeses, and the pouring method was applied to coat with the spice mixture. Coated cheeses were dried for 12 h at +4 °C and then packed in vacuum-wrapped bags (OPACK, QZ 1000 MAXI, OPAK Machine Mold Packaging Automation Ind. & Trade Ltd. Co., Esenyurt, Turkey), then stored for 90 days at 4 ± 2 °C and 85% relative humidity. Cheese production and coating were performed in three repetitions (Figure 2A–C).

##### Weight Loss

To determine the effect of coating material on cheese weight loss, the methods of Sarıoğlu & Öner [30] and Kalkan [50] were modified. The first weighing was performed after pre-drying (12 h at +4 °C), and the second after 1 day of storage at 4 ± 2 °C. The weight loss of the cheeses is given as a percentage.

##### Textural Analysis

The texture profile analyses of the cheeses were performed using a TA.XT2; Texture Analyzer (Stable Micro Systems, TA.XT2, Godalming, UK). The cheeses were cut into cylinders with a diameter of 36 mm and a length of 25 ± 0.5 mm. Analysis conditions: P36R aluminum cylinder probe (diameter 36 mm); initial test speed, 1 mm/s; test speed, 2 mm/s; final test speed, 5 mm/s; compression ratio, 20%; holding time, 5 s; trigger force, 5 g. The obtained data were calculated using Texture Expert Exceed Version 2V3 (Stable Micro Systems, 1998) [57,58].

##### Microbiological Analyses Applied to the Cheeses

For the purpose of determining the microbiological properties of the cheeses, samples were analyzed both with the edible film coating material and without it. The control cheese (A) was analyzed directly. The coated cheeses were divided into edible-film-coated cheese (B), cheese with edible film coating removed (B-CR), cheese with edible film and spice mixture coating (C), and cheese with edible film + spice mixture coating removed (C-CR). Microbiological analysis was performed for each production run with three replicates.

The TS EN ISO 707 standard was followed for sample collection [59]. Ten grams of each cheese sample was mixed with 0.1% (*w*/*v*) sterile peptone water, and suitable dilutions were prepared. The pour-plating method was applied in the analysis. Yeast mold counts were determined using PDA, total aerobic mesophilic bacterial counts using PCA, and lactic acid bacterial counts using MRS medium. The yeast mold counts and total aerobic mesophilic bacteria in the cheese samples were determined according to the method specified by Harrigan and McCance [60], and the number of lactic acid bacteria was determined according to the method specified by Harrigan [61].

#### 2.2.4. Statistical Analysis

The Response Surface Methodology (RSM) analyses and cheese production experiments were carried out in three replicates (n = 3). The RMS was applied using Design Expert 6.02 software to optimize the formulation parameters of edible film and spice mixtures. Model adequacy was evaluated based on R^2^, adjusted R^2^, and lack-of-fit tests, and three-dimensional response surface and contour plots were generated to visualize factor interactions. Cheese production was performed according to a Completely Randomized Design (CRD). To evaluate the differences between the samples, a one-way analysis of variance (ANOVA) was conducted using the SPSS 9.0 software package. Two confidence levels of 99% (*p* < 0.01) were considered for statistical significance. Tukey’s test was applied to determine the significant differences among the obtained data [62].

## 3. Results and Discussion

### 3.1. Characterization and Optimization of Edible Films with RSM

KG and WPI concentrations were selected as independent variables (X) in the preparation of the edible film coatings, and the WVP, E, and TS analysis results were taken as dependent variables (Y). The data acquired from the latter are given in Table 2.

#### 3.1.1. Water Vapor Permeability of Edible Films

In foods, water can cause microbiological, chemical, and textural deterioration. Therefore, water vapor permeability (WVP) is one of the most important properties of edible films; we determined that the WVP values of the films constructed in this study were between 4.50 G mm/m^2^·d·kPa and 7.05 G mm/m^2^·d·kPa (Table 2). In the literature, it is shown that polyamide, commonly used in cheese packaging, has a water vapor permeability of 0.5–10 G mm/m^2^·h·dPa, while polyethylene has a water vapor permeability of 0.5–2 G mm/m^2^·h·dPa [63]. Examining the R^2^ value of the variance analysis model, a 55% match between the experimental and estimated data was found (Table A1). The R^2^ value ranges from 0 to 1, and the further this value is from 1, the weaker the model’s predictive power becomes. Additionally, a large difference between the adjusted R^2^ and the R^2^ in the model, less than 0.2, is not desired [64,65]. The adequate precision value of the model was found to be 7.27. As this value was greater than 4, the design was considered to be usable [66]. Upon examining the variance analysis table for the model, the effect of edible film composition on water vapor permeability values was found to be significant (*p* < 0.05), as were the effects of WPI (X_1_) and KG (X_2_) quantity (*p* < 0.05).

The equation (Equation (3)) for the coded factors of the linear regression model, which describes the relationship between the independent variables (WPI and KG) for water vapor permeability, is given below.

WVP = +5.64 − 0.48 × X_1_ + 0.49 × X_2_ (3)

**Figure 3:**
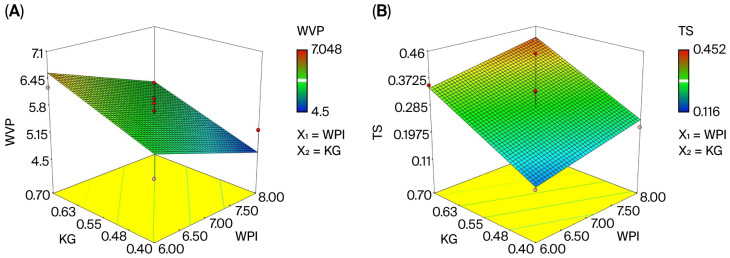

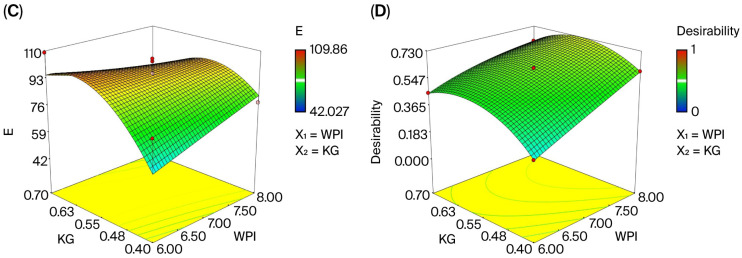
Three-dimensional response surface plots for the characterization and optimization of the edible film. (**A**) the effect of WPI and KG amount on WVP; (**B**) the effect of WPI and KG amount on TS; (**C**) the effect of WPI and KG amount on E; (**D**) desirability graph of WPI and KG.

The three-dimensional surface response graph illustrating the relationship between water vapor permeability and whey protein isolate (WPI) and konjac glucomannan (KG) concentrations is presented in Figure 3A, demonstrating that the concentrations of both affected permeability (*p* < 0.05). The literature reports that the water solubility coefficient of edible films decreases due to the formation of intramolecular disulfide bonds in whey protein [36]; however, the use of KG and WPI in the edible film matrix has a stabilizing effect on WVP by altering the film’s structural properties [26]. The findings of this study are consistent with those of Leuangsukrerk et al. In the model equation, it can be seen that the concentration of WPI has a negative effect on water vapor permeability, while the concentration of KG has a positive effect. Components with linear polymer structures like KG have poor water vapor permeability, while components with side chain polymer structures like proteins have higher permeability [28].

#### 3.1.2. Elongation Coefficient of Edible Films

The E value of the edible films ranged from 42.03% to 109.86% (Table 2). In the variance analysis, the effects of the model and of the amounts of WPI (X_1_) and KG (X_2_) on the elongation coefficient were not found to be significant (*p* > 0.05). This can also be seen in the three-dimensional response surface graph given in Figure 3B. As the concentration of KG in the edible film increased, the elongation coefficient increased slightly and then decreased. This can be explained by the increase in bond density in the polymer matrix; in hydrogel-based systems, increasing the polymer concentration can affect mechanical properties by increasing bond density [67]. It is thought that the change observed in the films’ E values was due to the increase in KG concentration in the matrix; first, the hydroxyl groups form bonds with the free-state amine groups in the environment, and then the bonds formed between the KG and themselves increase the bond density in the matrix. Leuangsukrerk et al. [26] also reported that lower WPI concentrations were associated with higher %E values in composite films composed of KG and WPI. In contrast, films containing only KG were reported to have lower elongation values compared to the KG-WPI blends. Similarly, Yoo and Krochta [68] reported that the combination of certain polysaccharides (e.g., methylcellulose, hydroxypropylmethylcellulose, and sodium alginate) with WPI had no significant effect on %E value.

#### 3.1.3. Tensile Strength of Edible Films

The TS values of the edible films varied between 0.12 MPa and 0.45 MPa (Table 2). In the variance analysis, the agreement between the experimental and estimated data was 64% (Table A2). It has been determined that the difference between the R2 and Adjusted R2 values of the model is less than 0.2. In addition, it was determined that the adequate precision value of the model was 8.13 and therefore suitable [66]. The analysis of variance demonstrated that the effect of the films on the tensile strength of the model was significant (*p* < 0.01). The impact of WPI (X_1_) quantity on tensile strength was not significant (*p* > 0.05), but the effect of WPI (X_2_) quantity was (*p* < 0.01).

The coded factor equation (Equation (4)) of the linear regression model defining the relationship between the tensile strength value and the independent variables is given below.

TS = +0.29 + 0.039 × X_1_ + 0.090 × X_2_ (4)

The increase in tensile strength with increasing KG content in the edible film composition is shown in Figure 3C. This change results from the film matrix gaining a more compact structure due to the interaction between the amino groups of the protein and the hydroxyl groups of KG [35]. Similarly, Yoo and Krochta [68] reported that increasing the polysaccharide content in films containing WPI improved tensile strength.

#### 3.1.4. Optimization of Edible Films

The Design Expert 6.01 package program was used to numerically optimize the films. Edible films have functions such as protecting the structural integrity of food against mechanical impacts, slowing down gas transfer and water vapor permeability, and being used as a carrier surface for protective additives such as antioxidants and antimicrobials coated on the food surface. Therefore, the parameters used to determine the physical/mechanical properties of the films were the permeability of oxygen, carbon dioxide, and water vapor [69], in addition to tensile strength and elongation properties, which are commonly used [70,71].

The basic function of packaging is to protect food against both physical effects and microbiological spoilage. Packaging for semi-hard cheeses must have very low permeability values for O^2^, CO^2^, water vapor, and odor, in addition to good mechanical properties [72]. Therefore, the minimum value of water vapor permeability and the maximum elongation coefficient and tensile strength values are used in the production of independent variables when optimizing edible films [69]. By applying the numerical optimization, we determined the optimal values to be 8.00% (*w*/*v*) WPI and 0.56% (*w*/*v*) KG at a 73% desirability score, 5.21 G mm/m^2^·d·kPa WVP, 96.39% E, and 0.34 MPa TS. In optimization, the desirability score is initially considered an indicator of how close the target is to being reached. This score ranges from 0 to 1 and is expressed as a percentage. The closer the desirability score is to 1 (100%), the more successful the optimization [44,73]. The desirability score determined in the current study indicates that the production of edible films with low water vapor permeability and good mechanical properties met 73% of the desired criteria. This result indicates that our optimization is compatible and can effectively prevent moisture loss in cheese, reduce the risk of deformations such as cracking and breaking that may occur after application, and strengthen the mechanical resistance to film surface adhesion.

#### 3.1.5. Microstructural Properties of Optimized Edible Film

**Figure 4:**
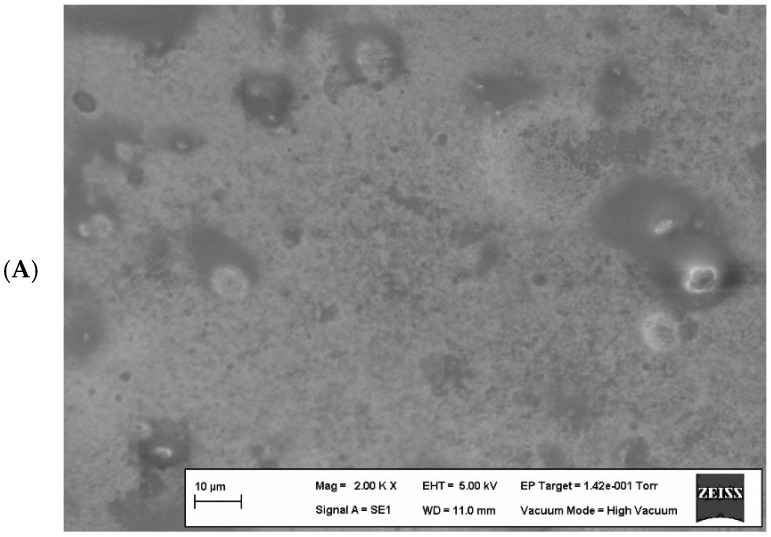

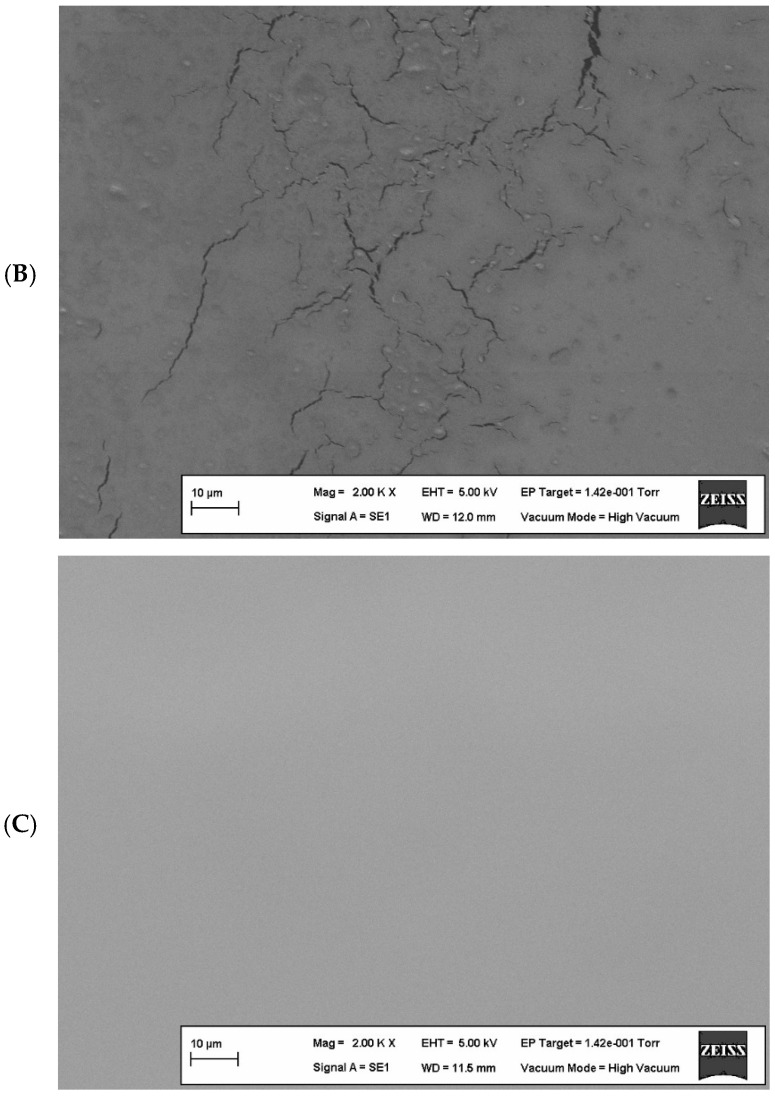
Scanning electron microscopy images of edible films. (**A**) contains 0.7% (*w*/*v*) KG, (**B**) contains 8% (*w*/*v*) WPI, and (**C**) contains 8% (*w*/*v*) WPI and 0.56% (*w*/*v*) KG.

Scanning electron microscope (SEM) images of the optimized edible films containing the upper limits of components (edible film containing 8.00% WPI and edible film containing 0.70% KG) are shown in Figure 4.

In Figure 4A, irregularities and microscopic bubbles can be observed on the surface of the edible film containing only KG. This is due to the weak interaction between glycerol and fatty acids in the film composition and the incompatibility between the polymers, causing a decrease in the force between molecules while affecting the film surface structure and water vapor permeability. Indeed, the literature reports that the plasticizing effect of glycerol in biopolymer films reduces the intermolecular forces along polymer chains and affects water vapor permeability [26,33,74,75].

When examining the edible film containing only WPI (Figure 4B), it was observed that although the addition of a plasticizer helped to form a homogeneous structure, there were micro-fractures in some areas. This can be explained by the limitation of polymer chain mobility due to strong interactions such as disulfide and hydrogen bonds formed between proteins in the film matrix [75].

Protein–polysaccharide complexes are formed through both covalent (disulfide bonds) and non-covalent interactions (hydrogen bonds and electrostatic attractions). In this case, where non-covalent interactions play a dominant role in the production of edible films, phase separation can be observed [76]. The interactions formed between the polysaccharide, protein, and plasticizer in edible film production support the film’s mechanical structure through covalent bonds, while hydrogen bonds increase the distance between protein polymer chains, allowing the film matrix to gain a flexible structure [33,35]. Therefore, the ratio of polysaccharides and proteins used in film production is critically important for morphology and mechanical properties. In Figure 4C, the homogeneity of the optimized edible film surface containing both WPI and KG is clearly visible. It has been stated in the literature that films with homogeneous surfaces generally have good mechanical properties [77].

### 3.2. Characterization and Optimization of Spice Mix by RSM

The amounts of thyme, rosemary, and red pepper were selected as independent variables (X) in the response surface method. The inhibition zones were considered the dependent variables (Y). The inhibition zones were formed by the extracts’ effect against *Aspergillus niger*, *Mucor* spp., and *Penicillium* spp., which are widely used in cheese production technology. The values determined as a result of our analyses are given in Table 4.

#### 3.2.1. Antifungal Effect of Spice Mixture Against *Aspergillus niger*

**Figure 5:**
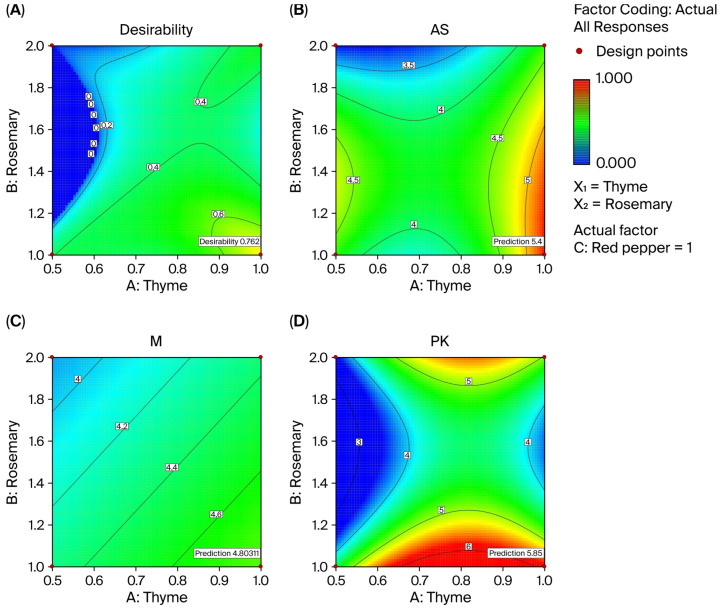
Projection graphics of the spice mixture optimization. (**A**) the overall desirability of antifungal activity as a function of thyme and rosemary concentrations; (**B**) antifungal activity against *Aspergillus niger*; (**C**) antifungal activity against *Mucor* spp.; (**D**) antifungal activity against *Penicillium* spp. (PK).

Table 4 shows that the antifungal inhibition zones of the spice mixtures against *Aspergillus niger* ranged from 5.40 mm to 3.10 mm. In the optimization study, the *p*-value of the model was determined to be 0.17, and the antifungal effect of the spice mixture against *Aspergillus niger* was found to be statistically insignificant (*p* > 0.05) (Figure 5B). It is believed that the observed change in the examples is due to random variation, and this is thought to be related to the type of spice, processing conditions, and extraction method [78]. Studies have shown that the antifungal effects of various capsicum species on *Aspergillus niger* can vary [79] and that thyme extract exhibits antifungal activity against *Aspergillus niger* MOM 05.11, while rosemary extracts do not have antifungal effects against this strain [80,81].

#### 3.2.2. Antifungal Effect of Spice Mixture Against *Mucor* spp.

The antifungal inhibition zones formed by spice mixtures against *Mucor* spp. ranged from 3.55 mm to 5.70 mm (Table 4). In the analysis of variance against *Mucor* spp., the model’s signal-to-noise ratio was found to be acceptable because its adequate precision value (5.21) was greater than 4 [66]. The R^2^ value of the variance analysis model was found to be 0.36, and the difference between the R^2^ and adjusted R^2^ values was determined to be less than 0.2. (Table A3 and Figure 5C). However, the variance analysis showed that the effect of the experimental model was not statistically significant (*p* > 0.05), meaning that the overall effect of the spice mixture on *Mucor* spp. could be due to random variation. In contrast, it showed that the effect of thyme (X1) on *Mucor* spp. was statistically significant (*p* < 0.05), demonstrating a significant antifungal effect against *Mucor* spp. In the literature, it has been reported that thymol, found in thyme essential oil, exhibits antifungal activity against molds [82]. This finding is also consistent with the study conducted by Klarić et al. [83] on the sensitivity of *Mucor* spp. to thyme essential oil. The equation for the coded factors of the linear regression model based on the antifungal effect of the independent variables against *Mucor* spp. is given below (Equation (5)).

Inhibition zones = +4.35 − 0.24 × X1 − 0.22 × X2 + 0.007921 × X3(5)

We found that the antifungal activity of the other spices used against *Mucor* spp. was statistically insignificant (*p* > 0.05). This may be due to differences in the concentrations or active components of the extracts used. Studies have shown that rosemary essential oil exhibits antifungal activity against *Aspergillus niger* ATCC 15475 at a concentration of 10 μg/mL, and against *Mucor pusillus* ATCC 16458 at a concentration of 20 μg/mL [39]. The findings regarding the antifungal effect of the spice mixtures obtained in the study against *Mucor* spp. are consistent with those reported in the literature.

#### 3.2.3. Antifungal Effect of Spice Mixture Against *Penicillium* spp.

The antifungal inhibition zones formed by spice mixtures against *Penicillium* spp. range from 3.50 mm to 5.85 mm (Table 4). When the variance analysis of the model was examined, it was determined that the signal loading was sufficient (adequate precision >4), but the *p*-value of the model was higher than 0.05 (Figure 5D). This was not statistically significant (*p* > 0.05) for the antifungal effect of the spice mixture against *Penicillium* spp. and is potentially associated with the bacterial species and the extraction method used. Similarly, the literature reports that rosemary and thyme extracts can have differing antifungal activities [78,84]. Felšöciová et al. [85] demonstrated that rosemary and thyme extracts exhibited different antifungal effects depending on the concentration and bacterial species used (for rosemary, *Penicillium brevicompactum* 0.75–2.25 mm, Penicillium citrinum 0.75–2.75 mm, and *Penicillium expansum* 0.75–0.25 mm; and for thyme, *Penicillium brevicompactum* 9.00–4.75 mm, *Penicillium citrinum* 14.50–4.75 mm, and *Penicillium expansum* 12.00–3.75 mm).

#### 3.2.4. Optimization of Spice Mixture

The Design Expert 6.01 software package was used for the optimization of the spice mixture, and the numerical optimization method was applied, aiming to maximize the antifungal effect of the spice mixture against *Aspergillus niger*, *Mucor* spp., and *Penicillium* spp. The literature reports that thyme, rosemary, and red pepper have antifungal effects against various types of mold [39,86,87,88]. Therefore, the maximum values of the independent variables for the formation of inhibition zones against *Aspergillus niger*, *Mucor* spp., and *Penicillium* spp. were selected to optimize the spice mixture. After optimization, it was determined that a spice mixture consisting of 1% thyme, 1% rosemary, and 1% red pepper showed an 84% desirability score for *Aspergillus niger* (5.40 mm), *Mucor* spp. (4.80 mm), and *Penicillium* spp. (5.85 mm). Projection plots for optimization are given in Figure 5A, indicating that the spice mixture approached the intended target of 84%. Theoretically, therefore, the antifungal effect of this mixture on these three types of mold reaches this level.

### 3.3. Cheese Analysis

#### 3.3.1. Weight Loss of Cheeses

The weight loss observed in cheese during drying is generally caused by the release of moisture into the environment. This also affects the dry matter content of the cheese [89]. Table 5 shows that the observed weight losses in the cheeses ranged from 7.26% to 9.07%. It was determined that the film coating caused a 1.81% reduction in weight loss, and this change was statistically significant (*p* < 0.01). The weight loss in the control (A) sample was higher than that of the edible-film-coated (edible-film-coated (B) and edible film + spice mixture-coated (C)) cheeses (Table 5). This is due to the edible film limiting the passage of water vapor. These findings are also supported by the literature and our water vapor permeability analysis. Tang et al. [90] reported that hydrogen bonds formed between soy protein isolate and carboxymethyl konjac glucomannan reduced the water vapor permeability of their films, Cerqueira et al. [19] found that the weight loss of emulsified edible coated cheeses was lower compared to uncoated cheeses, and Yılmaz & Dağdemir [89] stated that the application of edible film coating affected the moisture loss and dry matter content of cheeses. It has been determined that packaging materials containing polyethylene terephthalate and non-oriented polypropylene resulted in a weight loss of 3.08–6.09% for packaged cheeses during storage [91]. Additionally, Ruiz et al. [92] found that the weight loss of Mozzarella cheese packaged in polyurethane-based films was approximately 3% in the packaged cheese and 27% in the unpackaged cheese. These results indicate that a renewable film coating has a similar moisture barrier effect to conventional plastic packaging, with the limiting effect of moisture loss. However, in order to determine the effects of the identified reduction in weight loss, it was necessary to examine the textural properties of the cheeses.

#### 3.3.2. Textural Properties of Cheeses

Texture is an important parameter that affects the quality characteristics and consumer perception of cheese. The textural properties of the cheeses produced within this study are given in Table 5.

It was determined that the hardness values of the cheeses ranged from 4361.87 g to 3386.68 g, and the effect of the edible film coating material on the hardness value was statistically significant (*p* < 0.01). The coating process caused a decrease in the hardness value, with the highest hardness value shown by the control cheese (A). Cheese hardness is generally inversely proportional to moisture content. It has been stated in the literature that the changes caused by weight loss directly affect both the chemical composition and the textural properties of cheese [93].

The effect of edible films and spice coatings on the springiness value was found to be significant (*p* < 0.01). The highest flexibility value was seen in the control cheese (A). Generally, as the hardness of cheese increases, its elasticity value decreases; however, in semi-hard fresh cheeses, both hardness and elasticity can be high. In the literature, this has been found to be a result of the casein network structure formed during cheese production, contributing to the hardness of the cheese and the moisture in the cheese softening the texture [94,95].

Cohesiveness is a measure of the cheese’s tendency to stick together during compression and resist fragmentation [2,96]. The control cheese (A) had the highest cohesiveness value, while the other cheeses (B and C) had similar values (*p* < 0.01). In semi-hard cheeses, it has been reported that there is a positive relationship between the hardness value and the cohesiveness value [97].

Chewiness is one of the secondary textural parameters of cheese and varies depending on the values of hardness, elasticity, and stickiness [98]. The effect of the edible film and spice mixture on the chewability value was statistically significant (*p* < 0.01). Chewability value in hard and semi-hard cheeses shows a similar trend to hardness and cohesiveness [97]. Adhesion refers to the work required to overcome the attractive forces within the protein matrix [99].

The adhesiveness of cheese varies depending on the type of cheese and is determined by the food’s hardness and cohesiveness values [100]. The adhesiveness values of the cheeses in this study ranged between −2.62 and −3.58. In the literature, it is generally stated that as the hardness of hard and semi-hard cheeses increases, their adhesiveness value decreases [101]. This finding is consistent with the results of the current study.

We determined that the control cheese (A) had the highest gumminess value, while the chewiness values of the coated cheeses (B and C) were similar. Studies have reported that cheese with low moisture content has higher values for texture properties such as hardness, elasticity, cohesiveness, chewiness, and gumminess [98,100].

Finally, various packaging materials can be used in food packaging depending on the properties desired. Polyethylene–polyamide materials are preferred in food packaging to prevent oxygen, moisture, and aroma loss: polyethylene terephthalate is chosen for its high mechanical strength and low water vapor permeability, polyamide materials are selected for their high barrier properties and thermal resistance, and polypropylene lamination materials are preferred for their flexibility and sealing properties [102,103,104]. Based on the findings of the current study, we determined that the edible films and spice coating materials exhibited similar properties to those used in food industry packaging and contributed to the preservation of the cheese’s textural properties.

#### 3.3.3. Microbiological Properties of Cheeses

##### Number of Total Aerobic Mesophilic Bacteria

Film coatings affect the microbial stability of cheeses in addition to their physical and mechanical properties. The total aerobic mesophilic bacteria count of the cheeses varied between 4.55 log CFU/g and 6.70 log CFU/g (Table 6). It was observed that the total aerobic mesophilic bacteria counts in edible-film-coated cheeses (B) and edible film + spice mixture-coated cheeses (C) were higher than in the control cheese (A), while it was lower in cheeses from which the coating was removed (B-CR and C-CR). The application of edible film and spice coating did not have a statistically significant effect on the total aerobic microbial count (TAMB) (*p* > 0.05). In the literature, the total aerobic mesophilic bacteria (TAMB) count in Kaşar cheese was shown to range from 4.08 to 6.81 log CFU/g [105] and from 4.0 to 8.9 log CFU/g [106]. The value determined in our study is consistent with these reports and is quite close to the upper limit value (5 log CFU/g) specified by the International Commission on Microbiological Specifications for Foods [107].

The total number of bacteria on the cheeses increased during storage, and this change was found to be statistically significant (*p* < 0.01). The initial microbial population of cheeses is quite diverse and dynamic. Lactic acid bacteria (LAB) constitute a significant portion of the TAMB population in cheese [108]. Therefore, it is believed that a significant part of the change observed in the total aerobic mesophilic bacteria count is due to lactic acid bacteria. Indeed, the literature shows that the environmental acidity in cheese increases and the pH value decreases due to the ripening LAB population and chemical changes. This leads to the development of the LAB population and limits the growth of some acid-sensitive microorganisms [109]. The findings regarding TAMB in this study are similar to those of other studies [105,110].

##### Number of Lactic Acid Bacteria

Lactic acid bacteria are the most commonly used starter cultures in cheese production [111]. Table 6 shows that the number of lactic acid bacteria (LAB) in cheeses ranges from 3.05 log CFU/g to 5.23 log CFU/g (previous studies have determined that the number of LAB in Kashar cheese varies between 3.05 log CFU/g and 5.02 log CFU/g during storage [108]. Our findings are consistent with the findings of Öner et al. [112]. It was determined that the application of edible film coating had no significant effect on the number of LAB in the cheeses (*p* > 0.05). In contrast to our study, Ramos et al. [113] reported that the number of lactic acid bacteria in edible-film-coated cheese samples was higher compared to the uncoated control sample. The LAB counts of the cheeses increased during the storage period. This change was found to be statistically significant (*p* < 0.01). The literature highlights the ethics of LAB development, which plays an important role in the quality characteristics of cheese during storage [112,114,115]. Our findings reveal that the edible film and spice mixture played a role in the formation and preservation of cheese quality criteria by supporting the development of LAB, which is important for cheese technology.

##### Yeast and Mold Counts

In addition to LAB, yeasts and molds can also be found in cheeses. Yeasts play a significant role in the spoilage of dairy products due to their low water activity, high salt concentration, low pH value, ability to grow at low temperatures, specific enzyme activity, and nutritional requirements [116]. The formation of unwanted mold in dairy products poses a risk to human health and causes economic losses [117,118]. It was determined that the number of yeasts and molds formed during the storage period of the cheese varied between 1.27 log CFU/g and 5.12 log CFU/g (Table 6). The effect of the edible film coating and spice mixture on the amount of yeast and mold in cheese was found to be statistically significant (*p* < 0.01). The lowest yeast and mold counts in cheeses were found in the cheese coated with the edible film + spice mixture (C), and the highest in the control cheese (A). This result can be attributed to the antifungal properties of the spices added to the coating material and the permeability of the edible film coating. Indeed, the literature has shown that thyme and rosemary essential oils have antifungal effects on molds in different proportions, and that edible film coatings limit the number of yeasts and molds in cheeses [89,119]. Additionally, we found that the yeast and mold counts of all the cheese samples (B, B-CR, C, and C-CR) were lower than those of the control cheese (A). These findings indicate that edible film coating and edible film + spice coating can significantly limit the growth of yeast and mold on cheeses [89,120]. Yeast and mold counts increased during storage, which was found to be statistically significant (*p* < 0.01). These findings are similar to the results of other studies [121,122]. As a result, the edible film and spice mixture applied to the cheese significantly inhibited mold growth on the cheese and contributed to extending the shelf life of the cheese.

## 4. Conclusions

In cheese production, it is important to preserve the characteristic properties of cheese and extend its shelf life. Accordingly, various means, such as vacuum and modified-atmosphere packaging, are employed toward this end. In this study, we aimed to develop an edible film and spice blend coating material that has functions similar to traditional packaging methods and reduces packaging waste and to examine the physical, textural, and microbiological properties of this coating material on cheese.

In this context, optimization was performed to produce edible films (8.00% (*w*/*v*) WPI and 0.56% (*w*/*v*) KG) and spice mixtures (1% thyme, 1% rosemary, and 1% red pepper), which were then applied to the cheese surface. The system developed in the study showed similar performance to some conventional packaging used in the food industry in terms of water vapor barrier, tissue protection, and antifungal activity.

It was determined that the edible film and spice mixture coating reduced weight loss in cheese by 1.81%, affected the textural properties of the cheese, supported the development of LAB, and limited the growth of yeast and mold. The results showed that edible film and spice coating could reduce cheese quality losses and extend shelf life.

The fact that the coating material produced in the study is suitable for food contact and supports the quality characteristics of cheese makes this application feasible for use in small and medium-sized cheese businesses. However, it is considered that expanding the experimental design, conducting additional repetitions, and eliminating statistical limitations would be beneficial, particularly to increase the reliability of textural and microbiological findings and demonstrate the widespread applicability of the practice in the cheese industry.

Future studies will focus on different cheese types and storage conditions, and the integration of various active components into the film matrix, providing a more comprehensive assessment of the method’s industrial applicability and sustainability.

## Figures and Tables

**Table 1:** Edible film experimental design.

Independent Variable	Code	−α	−1	0	+1	+α
WPI (%)	X_1_	5.59	6.00	7.00	8.00	8.41
KG (%)	X_2_	0.34	0.40	0.55	0.70	0.76
**No**	**X_1_**			**X_2_**		
1	7.00			0.76		
2	7.00			0.55		
3	5.59			0.55		
4	7.00			0.55		
5	7.00			0.55		
6	6.00			0.70		
7	7.00			0.34		
8	6.00			0.40		
9	8.00			0.40		
10	8.00			0.70		
11	7.00			0.55		
12	7.00			0.55		
13	8.41			0.55		

**Table 2:** Results for response surface methodology of edible film (n = 3).

No	WVP (G mm/m^2^·d·kPa)	E (%)	TS (MPa)
1	169.14 ± 4.033 ^a^	70.52 ± 5.421 ^e^	0.35 ± 0.018 ^b^
2	136.62 ± 0.696 ^b,c,d^	87.51 ± 1.598 ^c^	0.27 ± 0.008 ^c^
3	153.54 ± 4.741 ^a,b^	73.74 ± 1.058 ^d,e^	0.18 ± 0.007 ^d,e^
4	143.65 ± 6.594 ^b,c^	96.08 ± 1.062 ^b^	0.33 ± 0.026 ^b^
5	152.62 ± 3.324 ^a,b^	103.49 ± 3.099 ^a^	0.45 ± 0.009 ^a^
6	149.92 ± 1.984 ^a,b^	108.78 ± 1.056 ^a^	0.35 ± 0.014 ^b^
7	118.93 ± 5.568 ^d,e^	42.03 ± 1.897 ^g^	0.12 ± 0.012 ^f^
8	121.86 ± 2.184 ^c,d,e^	79.86 ± 0.910 ^d^	0.15 ± 0.025 ^e,f^
9	125.32 ± 6.698 ^c,d,e^	78.21 ± 1.990 ^d^	0.22 ± 0.013 ^d^
10	119.67 ± 2.476 ^d,e^	63.37 ± 1.208 ^f^	0.41 ± 0.018 ^a^
11	141.02 ± 2.429 ^b,c,d^	90.93 ± 1.029 ^b,c^	0.33 ± 0.018 ^b^
12	119.70 ± 4.551 ^d,e^	105.18 ± 2.942 ^a^	0.33 ± 0.020 ^b^
13	108.06 ± 4.091 ^e^	109.86 ± 1.089 ^a^	0.32 ± 0.017 ^b, c^

Water vapor permeability (WVP), elongation coefficient (E), and tensile strength (TS) of edible films. Different lowercase letters (e.g., a, b, c) within the same column indicate significant differences among film material (*p* < 0.05).

**Table 3:** Spice mixture experimental design.

Independent Variable	Code	−α	−1	0	1	+α
Thyme (%)	X_1_	0.33	0.50	0.75	1.00	1.17
Rosemary (%)	X_2_	0.66	1.00	1.50	2.00	2.34
Red pepper (%)	X_3_	0.66	1.00	1.50	2.00	2.34
**No**	**X_1_**		**X_2_**		**X_3_**	
1	1.00		2.00		2.00	
2	0.75		1.15		2.34	
3	1.00		1.00		1.00	
4	0.75		1.50		1.50	
5	0.75		1.50		1.50	
6	1.00		1.00		2.00	
7	0.33		1.50		1.50	
8	0.75		1.50		1.50	
9	1.17		1.50		1.50	
10	0.50		2.00		1.00	
11	0.75		1.50		0.66	
12	0.75		1.50		1.50	
13	0.75		0.66		1.50	
14	0.75		1.50		1.50	
15	0.50		1.00		1.00	
16	0.50		2.00		2.00	
17	1.00		2.00		1.00	
18	0.75		1.50		1.50	
19	0.50		1.00		2.00	
20	0.75		2.34		1.50	

**Table 4:** Results for response surface methodology of spice mixture (n = 3).

No	AS (mm)	M (mm)	P (mm)
1	4.05 ± 0.150 ^c,d,e^	4.40 ± 0.100 ^c,d,e,f^	4.80 ± 0.200 ^b,c^
2	4.20 ± 0.200 ^b,c,d^	4.40 ± 0.100 ^c,d,e,f^	5.00 ± 0.500 ^b^
3	5.40 ± 0.050 ^a^	5.70 ± 0.300 ^a^	5.85 ± 0.050 ^a^
4	3.70 ± 0.300 ^d,e^	4.70 ± 0.200 ^b,c^	4.65 ± 0.050 ^b,c,d^
5	3.55 ± 0.350 ^e,f^	4.00 ± 0.000 ^f,g,h^	3.95 ± 0.050 ^e,f^
6	4.65 ± 0.150 ^b^	4.90 ± 0.100 ^b^	4.65 ± 0.150 ^b,c,d^
7	4.00 ± 0.300 ^c,d,e^	3.55 ± 0.050 ^h^	3.50 ± 0.200 ^f^
8	3.75 ± 0.500 ^d,e^	4.65 ± 0.150 ^b,c,d^	4.90 ± 0.000 ^b^
9	4.30 ± 0.100 ^b,c^	4.35 ± 0.150 ^c,d,e,f^	4.75 ± 0.250 ^b,c^
10	3.10 ± 0.100 ^f^	4.15 ± 0.050 ^e,f,g^	3.50 ± 0.100 ^f^
11	4.65 ± 0.150 ^b^	4.30 ± 0.200 ^c,d,e,f^	4.75 ± 0.050 ^b,c^
12	4.20 ± 0.100 ^b,c,d^	4.10 ± 0.10 ^e,f,g^	4.35 ± 0.050 ^c,d,e^
13	4.30 ± 0.000 ^b,c^	4.20 ± 0.000 ^d,e,f,g^	4.55 ± 0.150 ^b,c,d^
14	4.00 ± 0.100 ^c,d.e^	4.65 ± 0.150 ^b,c,d^	4.90 ± 0.100 ^b^
15	4.30 ± 0.100 ^b,c^	4.10 ± 0.100 ^e,f g^	4.50 ± 0.000 ^b,c,d^
16	3.95 ± 0.050 ^c,d,e^	3.95 ± 0.150 ^f,g,h^	4.65 ± 0.150 ^b,c,d^
17	4.35 ± 0.050 ^b,c^	3.75 ± 0.050 ^g,h^	4.95 ± 0.150 ^b^
18	3.15 ± 0.050 ^f^	4.50 ± 0.200 ^b,c,d,e^	4.20 ± 0.000 ^d,e^
19	3.85 ± 0.250 ^c,d,e^	4.65 ± 0.350 ^b,c,d^	4.65 ± 0.250 ^b,c,d^
20	4.10 ± 0.000 ^c,d^	4.20 ± 0.000 ^d,e,f,g^	3.90 ± 0.100 ^e,f^

AS: Inhibition zones demonstrating antifungal effect against *Aspergillus niger*. M: Inhibition zones demonstrating antifungal effect against *Mucor* spp. P: Inhibition zones demonstrating antifungal effect against *Penicillium* spp. Different lowercase letters (e.g., a, b, c) within the same column indicate significant differences among spice mixtures (*p* < 0.05).

**Table 5:** Weight loss and textural properties of cheeses (n = 3).

Sample	Weight loss (%)	Hardness (g)	Springiness	Cohesiveness	Chewiness	Adhesiveness	Gumminess
A	9.07 ± 0.126 ^a^	4361.87 ± 125.470 ^a^	0.99 ± 0.006 ^a^	0.90 ± 0.006 ^a^	4591.15 ± 69.525 ^a^	−2.62 ± 0.186 ^a^	4762.25 ± 110.826 ^a^
B	7.26 ± 0.271 ^b^	3386.68 ± 151.420 ^c^	0.93 ± 0.025 ^b^	0.88 ± 0.010 ^c^	4457.50 ± 67.185 ^b^	−3.57 ± 0.031 ^b^	4618.09 ± 41.536 ^b^
C	7.45 ± 0.489 ^b^	3435.32 ± 166.782 ^b^	0.93 ± 0.035 ^b^	0.89 ± 0.006 ^b^	3887.12 ± 31.041 ^c^	−3.58 ± 0.050 ^b^	4630.86 ± 50.643 ^b^

A: Control. B: Edible-film-coated. C: Edible film + spice mixture-coated. Different lowercase letters (e.g., a, b, c) within the same column indicate significant differences among coating treatments (*p* < 0.01). Adhesiveness values are expressed in negative values, indicating the direction of force.

**Table 6:** Microbiological properties of cheeses (log CFU/g) (n = 3).

		Storage (Day)			
Sample		1	15	45	90
Total aerobic mesophilic bacteria	A	5.07 ± 0.329 ^A^	5.66 ± 0.189 ^B^	6.19 ± 0.103 ^C^	6.58 ± 0.012 ^D^
	B	5.13 ± 0.587 ^A^	5.84 ± 0.465 ^B^	6.28 ± 0.099 ^C^	6.64 ± 0.076 ^D^
	B-CR	4.95 ± 0.062 ^A^	5.58 ± 0.410 ^B^	6.19 ± 0.178 ^C^	6.49 ± 0.065 ^D^
	C	5.38 ± 0.098 ^A^	5.75 ± 0.037 ^B^	6.24 ± 0.042 ^C^	6.70 ± 0.114 ^D^
	C-CR	5.07 ± 0.106 ^A^	5.59 ± 0.180 ^B^	5.93 ± 0.148 ^C^	6.50 ± 0.062 ^D^
Lactic acid bacteria	A	3.05 ± 0.041 ^D^	4.02 ± 0.062 ^C^	4.60 ± 0.026 ^B^	5.20 ± 0.021 ^A^
	B	3.07 ± 0.024 ^D^	4.06 ± 0.040 ^C^	4.70 ± 0.039 ^B^	5.23 ± 0.033 ^A^
	B-CR	3.07 ± 0.021 ^D^	4.05 ± 0.052 ^C^	4.68 ± 0.022 ^B^	5.21 ± 0.025 ^A^
	C	3.05 ± 0.029 ^D^	4.04 ± 0.057 ^C^	4.50 ± 0.095 ^B^	5.20 ± 0.051 ^A^
	C-CR	3.06 ± 0.041 ^D^	4.05 ± 0.045 ^C^	4.65 ± 0.037 ^B^	5.21 ± 0.025 ^A^
Yeast and mold	A	0.00 ± 0.000 ^a,D^	1.58 ± 0.298 ^a,C^	3.46 ± 0328 ^a,B^	5.12 ± 0.461 ^a,A^
	B	0.00 ± 0.000 ^a,C^	0.00 ± 0.000 ^b,C^	2.29 ± 0.198 ^b,B^	3.10 ± 0.078 ^b,c,A^
	B-CR	0.00 ± 0.000 ^a,C^	0.00 ± 0.000 ^b,C^	2.34 ± 0.260 ^b,B^	3.30 ± 0.294 ^b,A^
	C	0.00 ± 0.000 ^a,C^	0.00 ± 0.000 ^b,C^	1.27 ± 0.193 ^b,B^	2.06 ± 0.150 ^c,A^
	C-CR	0.00 ± 0.000 ^a,C^	0.00 ± 0.000 ^b,C^	1.38 ± 0.268 ^b,B^	2.09 ± 0.172 ^c,A^

A: Control. B: Edible-film-coated cheese. B-CR: Cheese with edible film coating removed. C: Edible film + spice mixture-coated cheese. C-CR: Cheese with edible film + spice mixture coating removed. Different lowercase letters (e.g., a, b, c) within the same column indicate significant differences among coating treatments (*p*< 0.01). Different uppercase letters (e.g., A, B, C) within the same row indicate significant differences among storage time (*p*< 0.01).

## Data Availability

The original contributions presented in this study are included in the article; further inquiries can be directed to the corresponding author.

## Appendix A

**Table A1:** Analysis of variance results for water vapor permeability of edible films.

Source	Sum of Squares	Mean of Squares	F-Value	*p*-Value
Model	3.69	1.84	6.06	0.02 *
X_1_	1.80	1.80	5.92	0.04 *
X_2_	1.88	1.88	6.19	0.03 *
Lack of fit	2.02	0.34	1.32	0.42
R^2^	0.55			
Adjusted R^2^	0.46			
Adequate precision	7.27			

* significant at 5%.

**Table A2:** Analysis of variance results for the tensile strength of edible films.

Source	Sum of Squares	Mean of Squares	F-Value	*p*-Value
Model	0.08	0.04	8.73	0.01 *
X_1_	0.01	0.01	2.78	0.13
X_2_	0.06	0.06	14.68	0.00 *
Lack of fit	0.03	0.00	1.02	0.52
R^2^	0.64			
Adjusted R^2^	0.56			
Adequate precision	8.13			

* significant at 1%.

**Table A3:** Variance analysis of the antifungal effect of the spice mixture against *Mucor*.

Source	Sum of Squares	Mean of Squares	F-Value	*p*-Value
Model	1.47	0.49	3.04	0.06
X_1_	0.77	0.77	4.80	0.04 *
X_2_	0.68	0.68	4.26	0.06
X_3_	0.00	0.00	0.01	0.94
Lack of fit	2.10	0.19	2.04	0.22
R^2^	0.36			
Adjusted R^2^	0.24			
Adequate precision	5.21			

* significant at 5%.
